# Supplementary material for: Structure basis for single-strand nucleic acid targeting by IscB and variants
Source: bioRxiv. 2026 Mar 4:2026.03.03.709405. Preprint. [Version 1] doi: 10.64898/2026.03.03.709405 (PMC12991159; doi:10.64898/2026.03.03.709405)
Supplement: 1 [file NIHPP2026.03.03.709405V1-supplement-1.pdf]

bioRxiv preprint doi: https://doi.org/10.64898/2026.03.03.709405; this version posted March 4, 2026. The copyright holder for this preprint (which was not certified by peer review) is the author/funder, who has granted bioRxiv a license to display the preprint in perpetuity. It is made available under aCC-BY 4.0 International license.

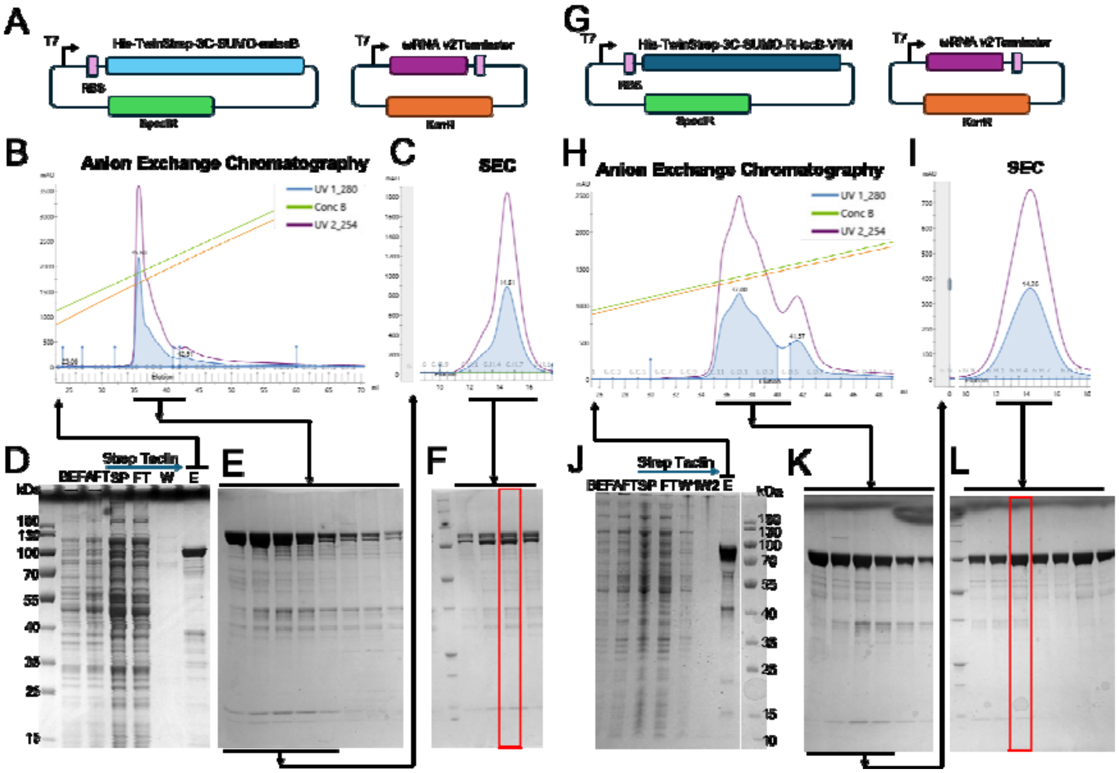

Supplementary Figure 1. Reconstitution of OgeuIscB-ωRNA RNPs. (A-F) Workflow of engineered IscB RNP purification. (A) Co-expression scheme of enIscB and ωRNA plasmids. (B) Elution profile of the enIscB-ωRNA RNP on anion exchange chromatography. (C) Elution profile of enIscB-ωRNA RNP on size-exclusion chromatography (SEC). (D) SDS-PAGE analysis of the Strep-tactin purified enIscB-ωRNA RNP. Before induction (BEF), after induction (AFT), lysate supernatant (SP), strep resin flow thru (FT), wash(W), and elution (E). (E) SDS-PAGE of anion exchange peak fractions. (F) SDS-PAGE of SEC peak fractions. (G-M) Workflow of R-IscB-VR4 RNP purification. (G) Co-expression scheme of R-IscB-VR4 and ωRNA plasmids. (H) Elution profile of the R-IscB-VR4-ωRNA RNP on anion exchange chromatography. (I) Elution profile of R-IscB-VR4-ωRNA RNP on size-exclusion chromatography (SEC). (J) SDS-PAGE analysis of the Strep-tactin purified R-IscB-VR4-ωRNA RNP. Before induction (BEF), after induction (AFT), lysate supernatant (SP), strep resin flow thru (FT), Dnase I wash (W1), wash2 (W2), and elution (E). (K) SDS-PAGE of anion exchange peak fractions. (L) SDS-PAGE of SEC peak fractions.

bioRxiv preprint doi: https://doi.org/10.64898/2026.03.03.709405; this version posted March 4, 2026. The copyright holder for this preprint (which was not certified by peer review) is the author/funder, who has granted bioRxiv a license to display the preprint in perpetuity. It is made available under aCC-BY 4.0 International license.

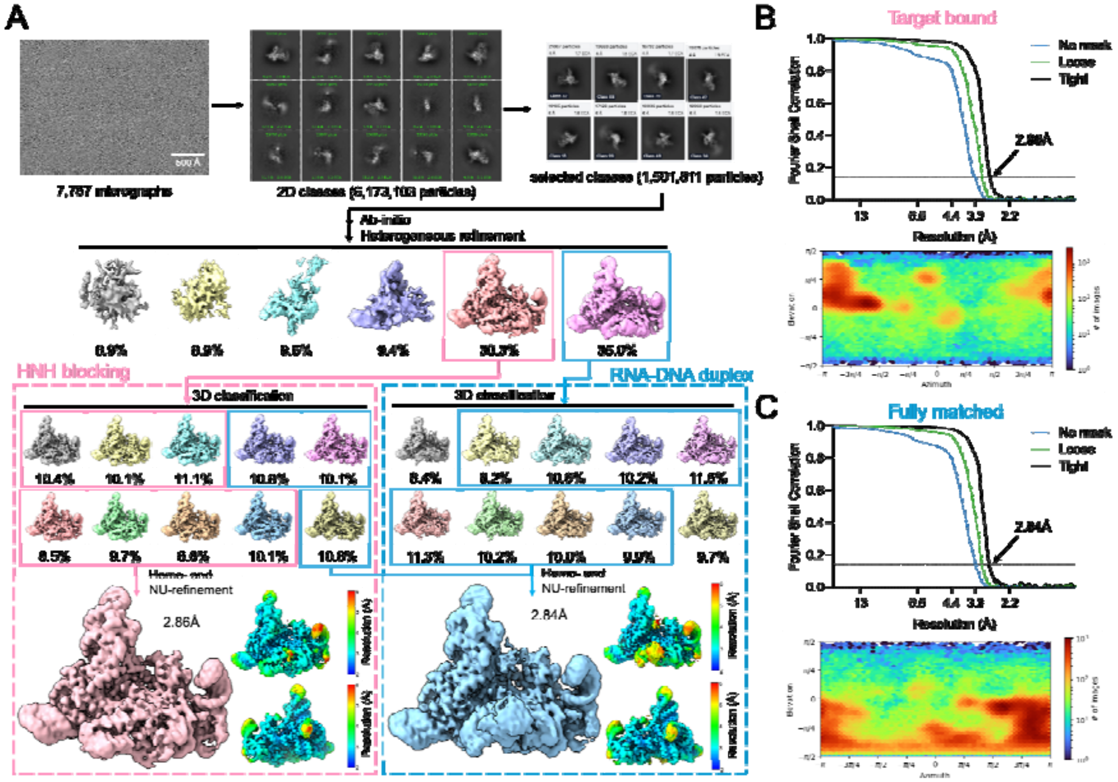

Supplementary Figure 2. Cryo-EM single particle reconstruction of enIscB-ωRNA-ssDNA complex. (A) Workflow of the cryo-EM image processing and 3D reconstruction for the enIscB-ωRNA-ssDNA complex. 3D classification was used to separate and reassign the particles to two states. (B-C) Top: Fourier Shell Correlations (FSC) of enIscB-ωRNA-ssDNA complex reconstruction with the gold-standard cutoff (FSC = 0.143) marked with a dotted line for (B) target bound state and (C) fully matched state. Bottom: Direction distribution plot for each state, respectively.

bioRxiv preprint doi: https://doi.org/10.64898/2026.03.03.709405; this version posted March 4, 2026. The copyright holder for this preprint (which was not certified by peer review) is the author/funder, who has granted bioRxiv a license to display the preprint in perpetuity. It is made available under aCC-BY 4.0 International license.

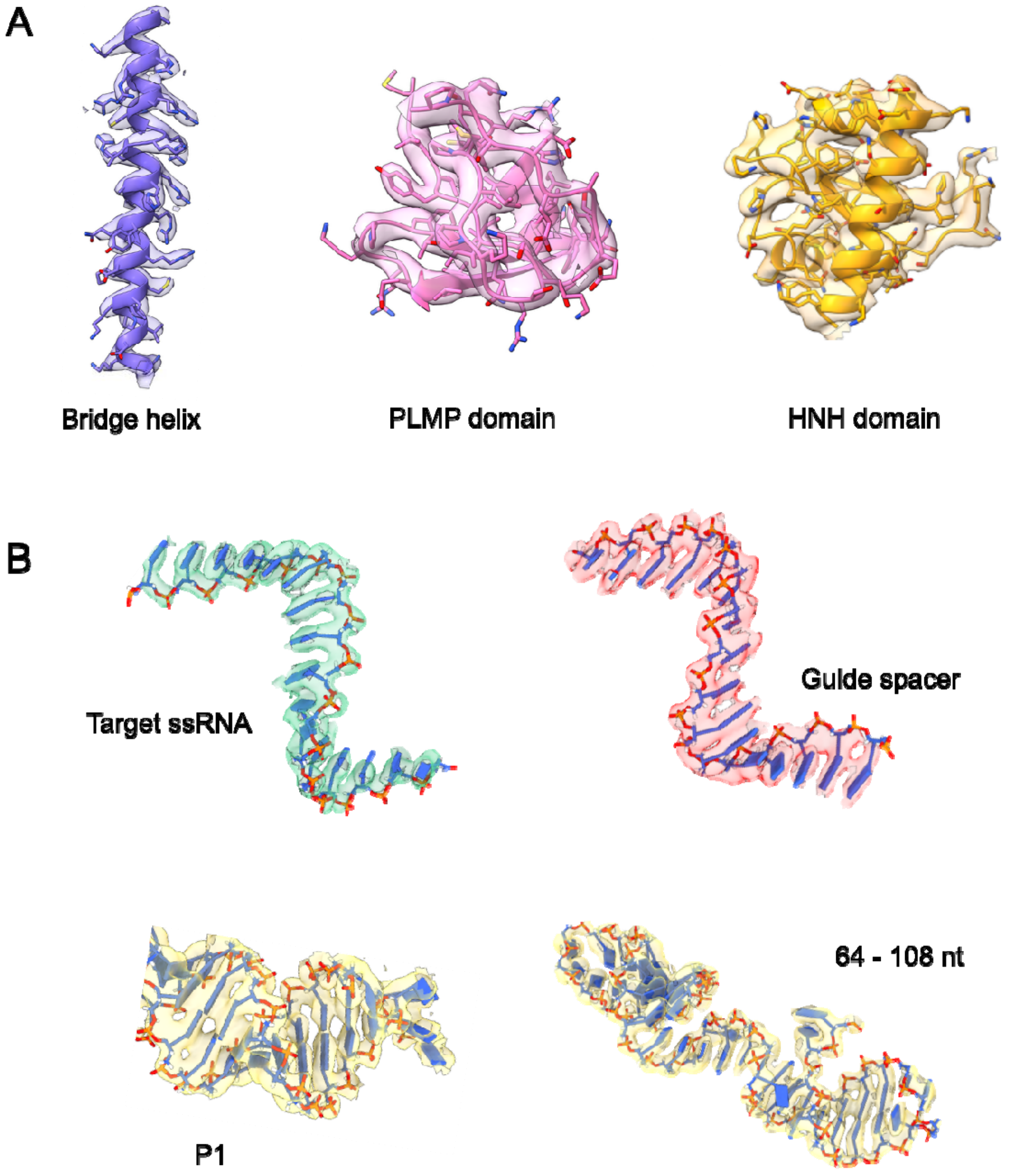

Supplementary Figure 3. Representative local map density for the different functional states. (A) EM densities for representative protein regions inside R-IscB-VR4-ωRNA-ssRNA complex. (B) EM densities for the target ssRNA strand and RNA regions inside the R-IscB-VR4-ωRNA-ssRNA complex.

bioRxiv preprint doi: https://doi.org/10.64898/2026.03.03.709405; this version posted March 4, 2026. The copyright holder for this preprint (which was not certified by peer review) is the author/funder, who has granted bioRxiv a license to display the preprint in perpetuity. It is made available under aCC-BY 4.0 International license.

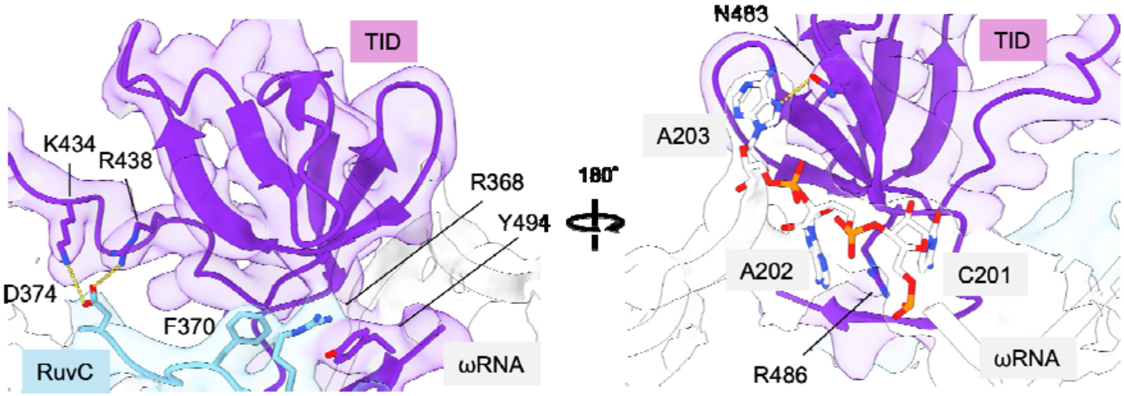

Supplementary Figure 4. Zoom-in views show the interactions between TID and adjacent RuvC domain and ωRNA, including salt bridge, cation-π stacking and hydrogen bonds.

bioRxiv preprint doi: https://doi.org/10.64898/2026.03.03.709405; this version posted March 4, 2026. The copyright holder for this preprint (which was not certified by peer review) is the author/funder, who has granted bioRxiv a license to display the preprint in perpetuity. It is made available under aCC-BY 4.0 International license.

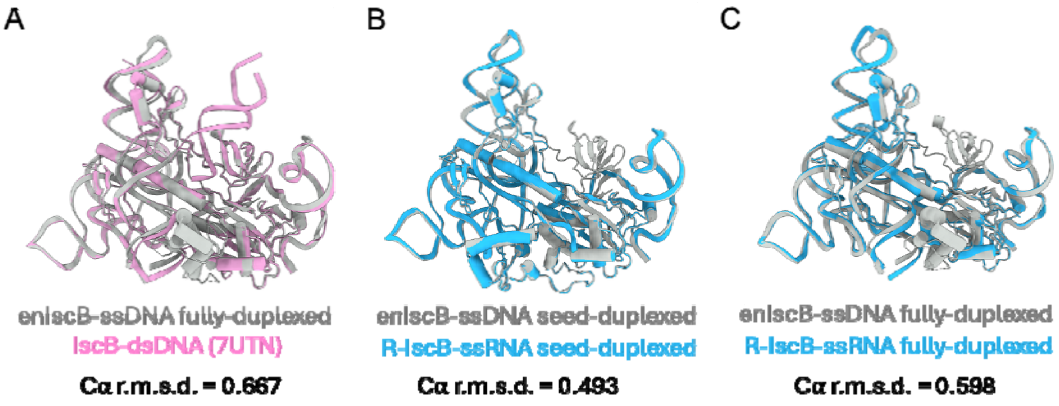

Supplementary Figure 5. Structure comparison of IscB and its variants. (A) Overlay of enIscB-ssDNA fully-duplexed state and previously reported IscB-dsDNA (PDB: 7UTN). (B) Overlay of enIscB-ssDNA and R-IscB-VR4-ssRNA in the seed-duplexed state. (C) Overlay of enIscB-ssDNA and R-IscB-VR4-ssRNA in the fully-duplexed state.

bioRxiv preprint doi: https://doi.org/10.64898/2026.03.03.709405; this version posted March 4, 2026. The copyright holder for this preprint (which was not certified by peer review) is the author/funder, who has granted bioRxiv a license to display the preprint in perpetuity. It is made available under aCC-BY 4.0 International license.

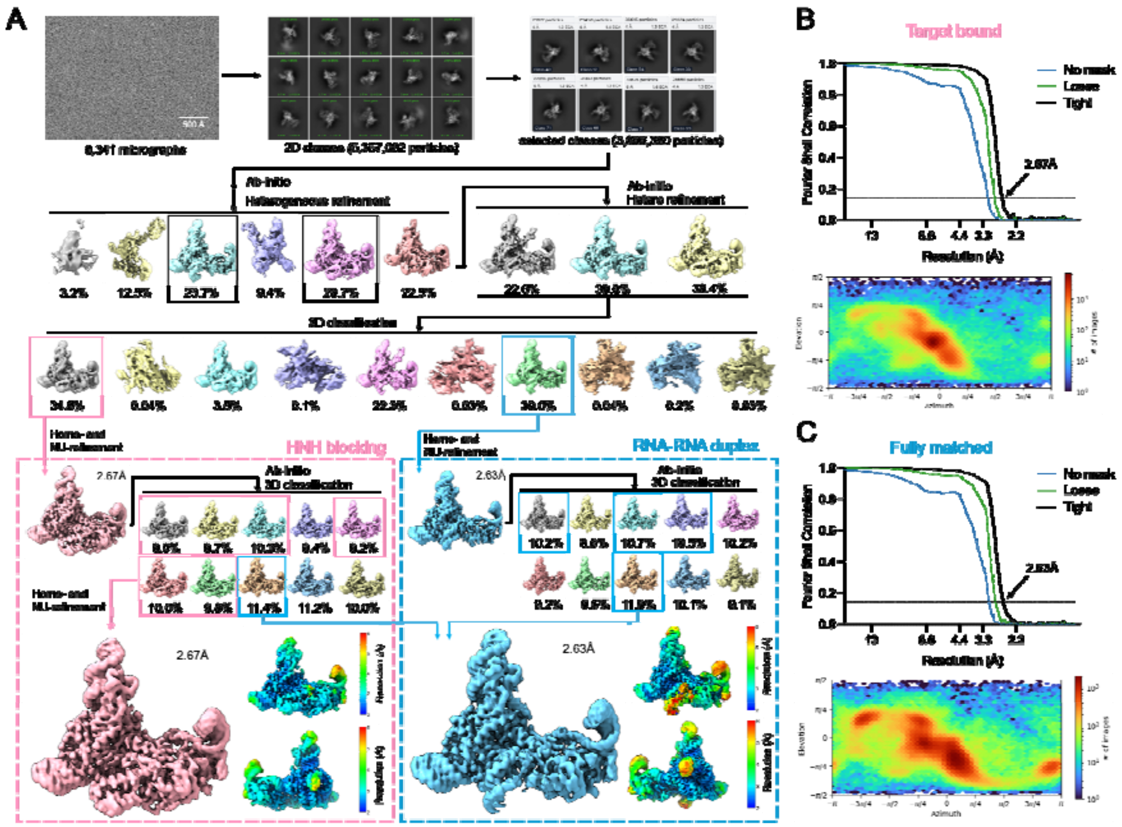

Supplementary Figure 6. Cryo-EM single particle reconstruction of R-IscB-VR4-ωRNA-ssRNA complex. (A) Workflow of the cryo-EM image processing and 3D reconstruction for the R-IscB-VR4-ωRNA-ssRNA complex. 3D classification was used to separate and reassign the particles to two states. (B-C) Top: Fourier Shell Correlations (FSC) of R-IscB-VR4-ωRNA-ssRNA complex reconstruction with the gold-standard cutoff (FSC = 0.143) marked with a dotted line for (B) target bound state and (C) fully matched state. Bottom: Direction distribution plot for each state, respectively.

bioRxiv preprint doi: https://doi.org/10.64898/2026.03.03.709405; this version posted March 4, 2026. The copyright holder for this preprint (which was not certified by peer review) is the author/funder, who has granted bioRxiv a license to display the preprint in perpetuity. It is made available under aCC-BY 4.0 International license.

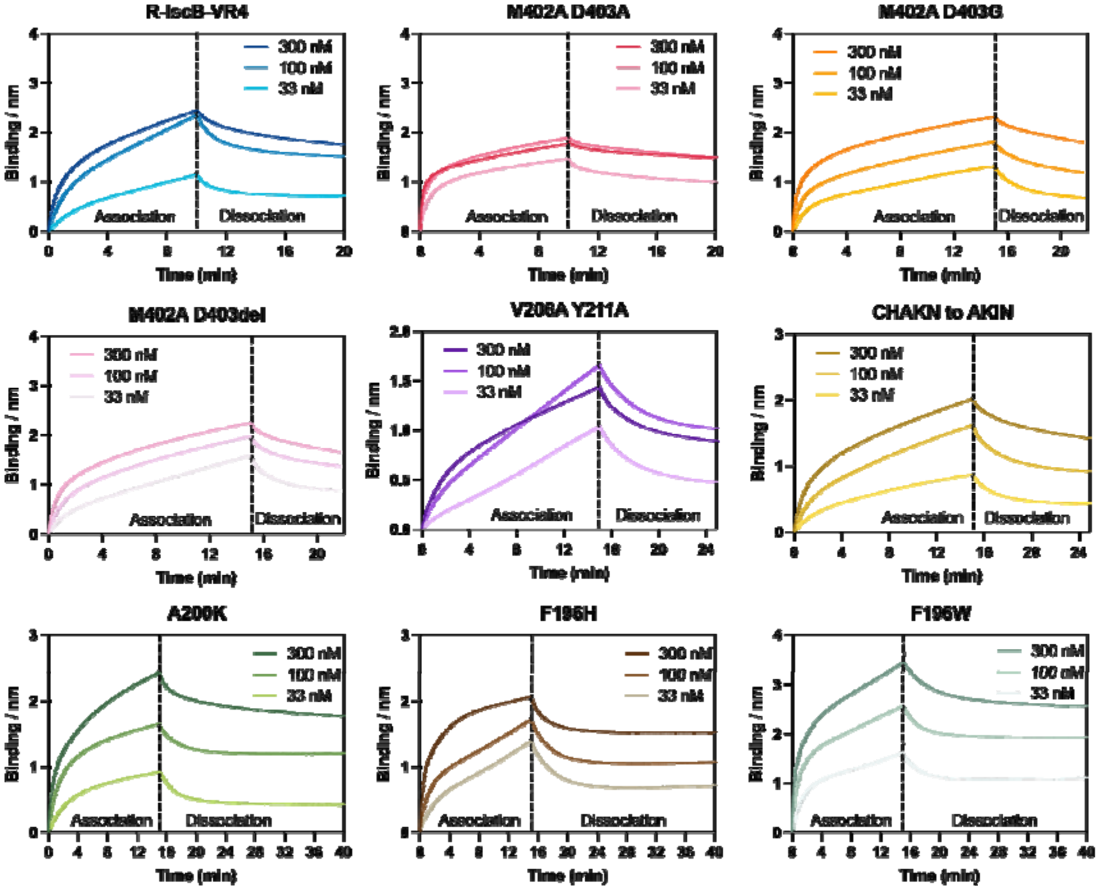

Supplementary Figure 7. Biolayer interferometry results of R-IscB-VR4 and its variants. Biotinylated ssRNA substrate was loaded onto streptavidin biosensor tips. Binding is represented by wavelength shift (Δλ, measured in nanometers) measured by Octet instrument.

bioRxiv preprint doi: https://doi.org/10.64898/2026.03.03.709405; this version posted March 4, 2026. The copyright holder for this preprint (which was not certified by peer review) is the author/funder, who has granted bioRxiv a license to display the preprint in perpetuity. It is made available under aCC-BY 4.0 International license.

Table S1. Cryo-EM data collection, refinement, and validation statistics

Name	8mut-HNH	8mut-Duplex	VR4-HNH	VR4-Duplex
PDB ID	11HU	11HR	11HX	11IC
EMDB ID	EMD-75703	EMD-75700	EMD-75706	EMD-75710
**Data collection and Processing (for each dataset)**				
Microscope	Krios G3i	Krios G3i	Krios G3i	Krios G3i
Voltage (keV)	300	300	300	300
Camera	K3	K3	K3	K3
Magnification	105,000x	105,000x	105,000x	105,000x
Pixel size at detector (Å/pixel)	0.4125	0.4125	0.4125	0.4125
Total electron exposure (e^{-}/Å^2)	50	50	50	50
Exposure rate (e-/pixel/sec)	15	15	15	15
Number of frames collected during exposure	40	40	40	40
Defocus range (μm)	-1.0 to -2.5	-1.0 to -2.5	-1.0 to -2.5	-1.0 to -2.5
Phase plate (if used)	N/A	N/A	N/A	N/A
-phase shift range (in degrees)				
-number of images per phase plate position				
Automation software (EPU, SerialEM or manual)	EPU	EPU	EPU	EPU
Tilt angle (if grid was tilted)	No	No	No	No
Energy filter slit width (if used)	15 eV	15 eV	15 eV	15 eV
Micrographs collected (no.)	7,757	7,757	8,341	8,341
Micrographs used (no.)	7,757	7,757	8,341	8,341
Total extracted particles (no.)	6,173,103	6,173,103	5,357,082	5,357,082
**For each reconstruction:**				
Refined particles (no.)	1,501,811	1,501,811	1,602,995	1,602,995
Final particles (no.)	291,738	293,636	319,480	328,824
Point-group or helical symmetry parameters	C1	C1	C1	C1
Estimated error of translations/rotations (if available)	N/A	N/A	N/A	N/A
Resolution (global, Å)				
FSC 0.5 (unmasked/masked)	3.0	3.0	2.8	2.8
FSC 0.143 (unmasked/masked)	2.9	2.8	2.7	2.6
Resolution range (local, Å)	2.0 – 10	2.0 – 10	2.0 – 10	2.0 – 10
Resolution range due to anisotropy (Å)	N/A	N/A	N/A	N/A
Map sharpening B factor (Å2) / (B factor Range)	-78	-78	-78	-78
Map sharpening methods	Global	Global	Global	Global

bioRxiv preprint doi: https://doi.org/10.64898/2026.03.03.709405; this version posted March 4, 2026. The copyright holder for this preprint (which was not certified by peer review) is the author/funder, who has granted bioRxiv a license to display the preprint in perpetuity. It is made available under aCC-BY 4.0 International license.

**Model composition (for each model)**				
Protein	495	476	433	334
Ligands	0	0	0	0
RNA/DNA	191	200	196	203
**Model Refinement (for each model)**				
Refinement package	PHENIX	PHENIX	PHENIX	PHENIX
- real or reciprocal space	real	real	real	real
- resolution cutoff	2.86	2.84	2.67	2.63
Model-Map scores				
-CC	0.89	0.87	0.88	0.82
-Average FSC	N/A	N/A	N/A	N/A
B factors (Å2)				
Protein residues	93.56	97.97	70.81	66.75
Ligands	N/A	N/A	N/A	N/A
RNA/DNA	95.02	97.13	74.09	68.19
RMSD from ideal values				
Bond lengths (Å)	0.004	0.004	0.005	0.003
Bond angles (°)	0.530	0.542	0.555	0.703
**Validation (for each model)**				
MolProbity score	1.64	1.51	1.37	1.45
CaBLAM outliers	0.0	0.0	0.0	0.0
Clashscore	7.67	8.12	6.68	4.61
Poor rotamers (%)	0.0	0.0	0.0	0.0
C-beta deviations	0.0	0.0	0.0	0.0
EMRinger score (if better than 4 Å resolution)	N/A	N/A	N/A	N/A
Ramachandran plot				
Favored (%)	96.55	97.66	98.84	96.67
Outliers (%)	0.0	0.0	0.0	0.0

bioRxiv preprint doi: https://doi.org/10.64898/2026.03.03.709405; this version posted March 4, 2026. The copyright holder for this preprint (which was not certified by peer review) is the author/funder, who has granted bioRxiv a license to display the preprint in perpetuity. It is made available under aCC-BY 4.0 International license.

Table S2. Kinetic measurements of R-IscB-VR4 RNP and its variants.

Mutations	K_d / pM	k_a (10^3 *M^{-1}*s^{-1})	k_{dis} (10^{-7} *1/s)
Parental R-IscB	22.53	9.454	2.13
M402A D403A	1.012	380.5	3.851
M402A D403G	21.73	7.387	1.918
M402A D403del	35.99	5.552	1.998
V206A Y211A	8.59 X 10^4	185.4	1.592*10^5
CHAKN to AKIN	19.25	10.62	2.044
A200K	23.08	8.309	1.918
F196H	8.82	17.23	1.520
F196W	22.55	5.433	1.225
